# Perioperative patient safety indicators—A Delphi study

**DOI:** 10.1111/jocn.17212

**Published:** 2024-05-17

**Authors:** Anette Nyberg, Maria Jirwe, Ami Fagerdahl, Volker Otten, Michael Haney, Birgitta Olofsson

**Affiliations:** ^1^ Department of Nursing Umeå University Umeå Sweden; ^2^ Department of Diagnostics and Intervention, Anaesthesiology and Intensive Care Medicine Umeå University Umeå Sweden; ^3^ Department of Health Sciences Swedish Red Cross University Huddinge Sweden; ^4^ Department of Neurobiology, Care Sciences and Society, Division of Nursing Karolinska Institutet Solna Sweden; ^5^ Department of Clinical Research and Education, Södersjukhuset Karolinska Institutet Solna Sweden; ^6^ Department of Diagnostics and Intervention, Orthopaedics Umeå University Umeå Sweden

**Keywords:** Delphi study, operating room, patient safety, perioperative care, process register, safety indicators

## Abstract

**Aim:**

To identify, define and achieve consensus on perioperative patient safety indicators within a Swedish context.

**Design:**

A modified Delphi method.

**Methods:**

A purposeful sample of 22 experts, all experienced operating room nurse specialists, was recruited for this study. A questionnaire was constructed incorporating statements derived from a preceding study. The experts were asked to rate the importance of each statement concerning patient safety during the perioperative phase. The data collection occurred through an online survey platform between November 2022 and April 2023. The CREDES checklist guided the reporting of this study.

**Results:**

The three‐round Delphi study resulted in consensus on 73 statements out of 103, encompassing 74% process indicators and 26% structure indicators. Key areas of consensus included the use of the Surgical Safety Checklist and optimizing the operating room environment.

**Conclusion:**

Consensus was reached on perioperative safety indicators, underscoring the intricate challenges involved in ensuring patient safety in the operating room. It emphasizes the important integration of both structure and process indicators for comprehensive safety assessment during surgical procedures. Recognizing the difficulty in measuring factors like teamwork and communication, essential for patient safety, the study offers practical guidance. It underlines a balanced approach and specific consensus areas applicable in clinical practice to enhance perioperative patient safety.

**Implications for the profession and patient care:**

This study provides concrete practice guidance and establishes a structured framework for evaluating perioperative care processes. It emphasizes the critical role of professionals having the necessary skills and being present during surgical procedures. Additionally, the study underscores the paramount importance of effective communication and teamwork within the operating room team, substantively contributing to overall patient safety enhancement.

**Impact:**

The study focused on addressing the challenge of ensuring patient safety in operating rooms, acknowledging the persistent complications related to surgery despite global efforts to eliminate avoidable harm in healthcare. Consensus was reached on 73 crucial indicators for perioperative patient safety, emphasizing a balanced approach integrating both process and structure indicators for a comprehensive assessment of safety during surgical procedures. The study has a broad impact on professionals and healthcare systems, providing concrete guidance for practice and offering a structured process for evaluating perioperative care.

**Reporting Method:**

The study is reported informed by ‘Guidance on Conducting and REporting DElphi Studies (CREDES) in palliative care: Recommendations derived from a methodological systematic review’.

**Patient or Public Contribution:**

No patient or public contribution.

**What does this paper contribute to the wider global community?**

- The perioperative safety indicators could be adopted and adapted by healthcare systems, contributing to a more comprehensive approach to perioperative safety.
- The research highlights the importance of a balanced integration of both structural and process indicators for a holistic evaluation of safety in surgical procedures.

## 1 INTRODUCTION

Operating rooms (ORs) are often described as a high‐risk environment for patients, with high rates of incidents and complications when patient safety is not prioritized. A valuable tool in this context is a process register, which can help in identifying and following up on clinical practice to improve patient safety in ORs. A comprehensive approach to follow‐up activities is vital, encompassing all measures affecting the patient outcome. This includes recognition of the different measures undertaken by operating room nurses, who consider patient safety as their most crucial nursing intervention (Alfredsdottir & Bjornsdottir, 2008).

## 2 BACKGROUND

Eliminating avoidable harm in healthcare remains a challenge worldwide (WHO, 2021). In high‐income countries, approximately one in ten patients experience adverse events during hospital care. Multiple and interconnected systems, such as intensive care units and ORs, are more disposed to mishaps (Reason, 1990). In Sweden, complications related to surgery account for 30% of events causing permanent patient harm (The National Board of Health and Welfare, 2023), often leading to financial compensation claims (LÖF, 2022).

Monitoring performance is fundamental for organisational improvement (Berwick, 1996). Nevertheless, healthcare workers face challenges in monitoring their daily performance due to a lack of information structures and consensus on practical monitoring methods (Institute of Medicine Committee on Quality of Health Care in A, 2001). Donabedian's systems‐based framework—structure, process and outcome—evaluates care quality (Donabedian, 1983). In this framework, structure refers to channels through which care is provided, the process to the actual care given and the outcome to the consequences of care (Campbell et al., 2003).

Currently, the assessment of outcome validity relies on a comprehensive approach (Donabedian, 1983). This encompasses various factors, including the evaluation of the patient, the provider, organisational structures and processes and the patient‐related outcomes. In healthcare, process measures are accepted as they show how caregivers actively improve processes and subsequently patient outcomes (Pronovost et al., 2004). Including process measures in routinely documented clinical data serves a dual purpose, reminding clinicians of correct procedures and reducing redundant data collection for quality assessment.

Process indicators convey key aspects of a given process, while structure indicators form benchmarks for setup and location (Donabedian, 1983). Profession‐specific safety assessments identify safe process elements (Pronovost et al., 2004) and support monitoring perioperative care safety. These indicators should be grounded in scientific research or expert consensus (Campbell et al., 2003), as the lack of such assessment tools obstructs the comprehensive evaluation of safety. This study encompasses both structure and process indicators.

Neglecting patient safety in perioperative care is linked with higher incident rates and complications (de Vries et al., 2008). Safety II focuses on smooth daily operations where safety is inherent in complex systems situations through joint efforts to adapt to changing situations and uncertainties, rather than the inherent state where nothing unpredicted occurs (Hollnagel et al., 2015). The focus should shift towards actively managing risks instead of striving for absolute safety, as in eliminating error and harm (Amalberti & Vincent, 2020).

Operating room nurse specialists (ORNs) are pivotal in improving patient safety by effectively managing risks throughout the perioperative process (Alfredsdottir & Bjornsdottir, 2008). This study focuses on practice based in Sweden, where ORNs are acknowledged as a necessary profession within the OR team for performing surgical procedures. International differences in ORNs' training, roles, and clinical responsibilities exist. In Sweden, becoming an ORN requires a 1‐year OR nursing specialist and an academic Master's program for a registered nurse (Swedish Ministry of Education and Research, 2023). Swedish ORNs are responsible for tasks including patient positioning, skin antisepsis and surgical draping, and instrument and implant management (Riksföreningen för operationssjukvård och Svensk sjuksköterskeförening. Kompetensbeskrivning avancerad nivå Specialistsjuksköterska inom operationssjukvård, 2023), while other countries have other professional categories managing some of these responsibilities.

Sweden has a tradition of collecting patient data for national clinical and quality registries (Aase & Schibevaag, 2017), managed within the regional healthcare authority system. These diagnosis or process registers are vital tools for tracking clinical practice improvements for enhanced patient safety. ORNs, with their expertise in perioperative patient care, should advocate for nursing data inclusion in existing databases for quality reporting (Westra & Peterson, 2016). Previous research suggests that improving ORNs' access to process information and feedback can enhance OR care quality (Ingvarsdottir & Halldorsdottir, 2018).

Efforts have previously been undertaken to define core elements of perioperative nursing, with a focus on ensuring patients' physiological and physical safety (Rauta et al., 2013). The Perioperative Nursing Data Set initiative aimed to establish essential data elements for perioperative care practice. Consistent definitions and terminology in practice documentation are crucial for defining fundamental aspects of perioperative nursing practice (Petersen & Kleiner, 2011). Clearly defined data variables are essential for meaningful practice comparison and evaluating the impact of perioperative nurses on patient outcomes.

In Sweden, there is limited agreement on significant perioperative patient safety indicators enabling feedback and follow‐up on the ORNs' different measures. With consensus on safety indicators derived from clinical practice, and applied to daily clinical practice, healthcare organizations could benefit from a comprehensive set of safety indicators and definitions of the perioperative process, focusing on patient safety. Therefore, as a first step towards developing valid safety indicators for perioperative nursing, this study aims to identify, define and reach a consensus on indicators of perioperative patient safety within a Swedish context through a Delphi process.

## 3 THE STUDY

### 3.1 Aim

To identify, define and reach a consensus on perioperative patient safety indicators.

### 3.2 Methods

#### 3.2.1 Study design

A modified Delphi study was conducted to gain agreement on perioperative patient safety indicators among a group of ORNs. Delphi survey is a technique for obtaining consensus from an expert panel about a subject commonly used within health and social sciences and is seen as a flexible approach to transforming individual opinions into a group consensus (Keeney et al., 2006). The reporting of this study was guided by Guidance on Conducting and REporting DElphi Studies (CREDES) in palliative care: Recommendations derived from a methodological systematic review (EQUATOR research reporting checklists) (Jünger et al., 2017).

#### 3.2.2 Participants

The participants for the expert panel were recruited through purposive sampling. Experts were defined as informed individuals (Keeney et al., 2011), ORNs with current knowledge of the research topic, and interest and involvement in the subject of interest. The inclusion criteria for experts were ORNs with clinical experience minimum of 2 years post‐training experience. The eligible ORNs for the study were identified and proposed by two authors (Anette Nyberg & Ami Fagerdah) through a national perioperative education and research network and the Swedish Operating Room Nurses Association. The rounds were conducted in Swedish, and the panel size was decided to be approximately 20, based on an attempt to find a balance between the ability to make a definite conclusion and the difficulty of managing a larger panel size (Keeney et al., 2011). Finally, recognized Delphi process expert helped the project coordinator (Anette Nyberg) interact with the panel members during the study, encouraging participation.

With a list of names and email addresses of the ORNs, an informational email requesting participation was sent. The information letter stated that the ORNs could also suggest additional experts to be approached. The invitation was sent to 35 ORNs, of which 20 agreed to participate. The first 20 experts contacted recommended two additional experts who decided to participate after receiving information about the study. In total, 22 ORNs were included in the expert group.

#### 3.2.3 Data collection

A questionnaire was constructed, and data were collected using an online survey platform (Artologik: Survey & Report, Artisan Global Media, Sweden). The questionnaire was pretested with four ORNs and two nurse researchers who did not participate in the study. After the pre‐test, some adjustments were made in wording where clarification was needed.

The level of consensus was decided before starting the study (Keeney et al., 2006). There is no clear general agreement on an optimal consensus level. In this study, a statement was considered to have reached a consensus if 80% or more of the experts rated a statement equally. This is to present the experts' responses as accurately as possible (Keeney et al., 2011). In addition, the statements to rate were derived from clinically active ORNs, who considered these statements essential to maintaining perioperative patient safety.

To start, a modified Delphi method was undertaken, using perioperative patient safety indicators identified in a previous study (Nyberg et al., 2021) as the basis for round one, unlike a classic Delphi where the first round is for generating ideas by the experts (Keeney et al., 2011). In the prior study, 89 statements were generated and divided into 14 groups. The groups are presented in Table 1.

**TABLE 1:** Statement groups as presented in the rounds.

Statement groups
For surgical planning to contribute to safe care
To complete the list of operations scheduled and not have to cancel any
To ensure that the work intraoperatively proceeds optimally
The flow of information contributes to increased safety during the procedure
Correct patient and correct side are ensured
The use of the Surgical Safety Checklist (SSC) contributes to improved patient safety during the procedure
The OR environment is optimized
Sterility during the procedure is ensured
To ensure that the right implant is operated in
For the management of indwelling urinary catheters (IDCs) to be safe in OR
To ensure the safe handling of surgical specimens during the procedure
To ensure safe positioning of the patient on the OR bed
To ensure that electrosurgical units do not injure the patient during the procedure
To ensure follow‐up and the possibility of improvement

*Note*: *N* = 14 groups.

The experts were asked to rate each statement according to how important they found it to be to obtain patient safety during surgery. They were asked to treat each statement individually and to indicate their level of agreement on the importance of each statement. A 5‐point Likert scale was used, with one for least important ‘not at all important’ and five for most important ‘very important’.

After each group of statements, there was an open dialogue box for the experts to use if they had suggestions for additional statements, on statement wording or if the statement needed clarification. This applied to all three rounds as well as that the experts were given 2 weeks to complete each survey and if they had not answered, a reminder email was sent on Days 3 and 6. For analysis, the results were exported to SPSS version 28 (IBM Corp., USA) after the survey concluded in all three rounds, with percentages calculated for each statement. In Rounds 1 and 2, the median was calculated for statements without consensus yet. After completing all three rounds means and standard deviations (SD) were calculated for all statements that reached consensus, irrespective of whether it occurred in Rounds 1, 2 or 3.

##### Round 1

In November 2022, the 22 experts received an email as a notification that the first questionnaire, Round 1, would be sent out from the survey platform shortly. In the questionnaire, the experts received instructions on how to complete the first round.

There were also demographic questions, including sex, age, years of experience as an ORN, central position at work, the proportion of clinical work, in which region and type of hospital the ORN works. From the 89 statements, 47 reached the pre‐set consensus level of 80%, and the experts added 14 new statements. The response rate in Round 1 was 100%.

##### Round 2

The second round took place at the beginning of February 2023, a month after the first round was completed. A new questionnaire was constructed with 56 statements, 42 were from the previous round where consensus had not been reached and 14 statements were generated by the experts' comments in Round 1. It was decided to exclude statements that had already reached a consensus in Round 1, to shorten the questionnaire (Keeney et al., 2011). Instead, a week before Round 2 was sent out, the experts received an email as a notification for the next round, including the results from Round 1 on the statements that had reached consensus. Furthermore, the same day the experts received the Round 2 survey, an email was sent with the remaining statements from Round 1 that had not reached a consensus, including their prior response and the group median. Round 2 also had a response rate of 100%.

##### Round 3

**FIGURE 1:**
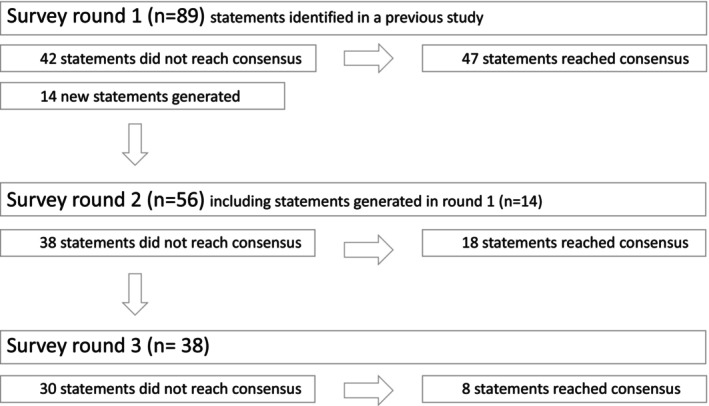
Statements in Delphi rounds.

The third round took place in March 2023. As in the previous round, a week before the start of Round 3, an email was sent to the experts with the results of Round 2. This was also a notification that Round 3, the final questionnaire, was coming. On the same day the Round 3 surveys were sent out, experts received an email containing the remaining statements from Round 2, including their responses and group median. The Round 3 surveys had 38 statements, all from the previous round where consensus had yet to be reached, and the response rate was 91% (Figure 1).

#### 3.2.4 Ethical consideration

The present study was approved by the Swedish Ethical Review Authority (reg. no. 2020‐00111) and follows the principles outlined in the Declaration of Helsinki (WMA, 2023). All responses were handled confidentially; only one person in the research group (Anette Nyberg) had a ‘key’ to connect a particular response to the individual expert's email address. The responses submitted in each round were linked to an assigned number generated in the survey platform. Although the experts might know each other, the rounds' results were presented at a group level, so they could not attribute responses to a specific expert. The experts' opinions remained strictly confidential (Keeney et al., 2011), also called quasi‐anonymity in Delphi method literature (Keeney et al., 2006).

## 4 RESULTS

There was a geographical spread among the experts in this study, consisting of ORNs in clinical practice, ORNs partly in management, including section management and care development and ORNs involved in nursing education and research. Table 2 provides detailed information on demographics.

**TABLE 2:** Demographics of the Delphi panel.

	Delphi round I and II (*n* = 22, response rate 100%)	Delphi round III (*n* = 20, response rate 91%)
Sex		
Female (%)	21 (96%)	19 (95%)
Male (%)	1 (4%)	1 (5%)
Age in years, mean (SD)	53 (8.4)	53 (8.2)
Experience as ORN in years, mean (SD)	25 (10.3)	24 (9.9)
Perioperative field, *n* (%)		
Clinical practice	5 (23%)	3 (15%)
Partly in nursing management	10 (45%)	10 (50%)
Nursing education and research	7 (32%)	7 (35%)

### 4.1 Round 1

In Round 1, 47 statements reached the pre‐set consensus level of 80%. The groups where most statements reached consensus in the initial round were ‘The use of the Surgical Safety Checklist (SSC) contributes to improved patient safety during the procedure by…’ and ‘The OR environment is optimized by…’, with each group having eight statements reaching consensus. However, these groups also had the largest number of statements initially to evaluate. Apart from the statement group labelled ‘To ensure that the right implant is operated in…’, two or more statements from all the other statement groups also reached consensus in Round 1.

Notably, eight out of the nine original statements in the ‘The use of the SSC contributes to improved patient safety during the procedure by…’ group reached consensus during the initial round, and the experts added one additional statement to this group. Similarly, in the ‘Correct patient and correct side are ensured by…’ statement group, both statements reached consensus, with experts adding a statement. However, 42 statements did not reach a consensus in Round 1 and the experts introduced 14 new statements based on their feedback and comments.

### 4.2 Round 2

In the second round, consensus was reached on an additional 18 statements, all of which were rated as ‘very important’. Consensus was reached on all statements within the ‘Correct patient and correct side is ensured by…‘ group of statements. However, no further statements reached consensus in the groups labelled ‘To ensure that the work intraoperatively proceeds optimally…‘, ‘The flow of information contributes to increased safety during the procedure by…‘ or ‘To ensure follow‐up and the possibility of improvement…‘. Additionally, there were no new statements introduced at this stage. Nevertheless, 38 statements still did not reach consensus in the second round.

### 4.3 Round 3

Eight statements were graded as ‘very important’ and reached a consensus in the third or final round. In total, 73 out of 103 statements reached consensus after three rounds, with a mean of 4.75–5.00 (SD 0.00–0.66), as shown in Table 3. Of the indicators, 19 (26%) were structure indicators and 54 (74%) were process indicators.

**TABLE 3:** Statements that reached consensus (≥80%) for all three rounds, including indicator type, mean, standard deviation and consensus level.

	Indicator type	Mean	SD	Consensus level
For surgical planning to contribute to safe care, it is necessary that				
The right implants are available	S	5.00	0.00	100%
The right *procedure card* has been chosen (procedure card here refers to the information in the planning, which intervention is to be performed, which instruments, implants and medical technical equipment are needed, how the patient is to be positioned, intraoperative drugs and planned surgery time)	P	5.00	0.00	100%
The right surgical instruments are available	S	4.95	0.02	96%
Changes in the surgical method are communicated and take place before preoperative preparations begin	P	4.82	0.39	81%
To complete the list of operations scheduled and not have to cancel any				
Implants need to be *checked the day before* and available before the start of surgery	S	4.91	0.29	91%
The right competence in the operating room (OR) team is needed for highly specialized interventions	S	4.91	0.29	91%
There need to be routines for preoperative preparations that are followed	S	4.86	0.35	86%
All preoperative examinations of the patient need to be performed before the patient arrives at the OR	S	4.86	0.35	86%
Instrument systems need to be clearly marked to avoid the wrong instruments being set up and thereby having consequences for the following patients	S	4.86	0.35	86%
Implant carts need to be checked before the start of setting up surgical instruments	S	4.82	0.39	82%
Surgical instruments need to be available for setting up before the start of the procedure	S	4.77	0.53	82%
To ensure that the work intraoperatively proceeds optimally				
All professionals in the OR team need to communicate	P	4.95	0.21	96%
All professionals in the OR team need to have the skills required	S	4.95	0.21	96%
All professionals in the OR team need to collaborate	P	4.91	0.29	91%
All professionals in the OR team have a common goal	P	4.91	0.29	91%
Operating room nurse needs to have time to check the patient's positioning before starting skin antisepsis and surgical draping	S	4.91	0.29	91%
All professionals required need to be in the OR during the procedure	S	4.85	0.35	86%
Operating room nurse needs to have time to read about the patient before starting preparations	S	4.80	0.41	80%
The flow of information contributes to increased safety during the procedure by				
Injuries to the patient being reported back to the OR staff	P	4.86	0.35	86%
Infections being fed back to the OR staff	P	4.82	0.39	82%
Correct patient and correct side are ensured by				
Confirming indicated side with medical records and x‐rays (when available) before anaesthesia is applied	P	5.00	0.00	100%
Verifying patient identity and confirming the indicated side with medical records and x‐rays (when available) when the patient enters the OR	P	4.95	0.21	96%
Communicating identity and indicated side with the patient before the procedure when possible	P	4.95	0.21	96%
The use of the Surgical Safety Checklist (SSC) contributes to improved patient safety during the procedure by				
A check that it is the correct patient	P	5.00	0.00	100%
Postoperative prescriptions are reviewed at check‐out, e.g. antibiotics are given at the right time	P	5.00	0.00	100%
A check that the correct instruments and implants are planned	P	4.95	0.21	96%
A verification that the surgical count is complete and accurate (instruments, etc.) before checking out	P	4.95	0.21	96%
Checking that it is the right intervention to be carried out	P	4.91	0.43	96%
Ensuring that everyone in the OR team feels that they have received the information they need before starting the procedure at the check‐in	P	4.82	0.66	91%
Everyone in the OR team is given the opportunity to participate in the check‐in	P	4.82	0.39	86%
Everyone in the OR team is given the opportunity to participate in the check‐out	P	4.82	0.50	86%
Everyone in the OR team respects the review of the SSC by not doing anything else at the same time	P	4.80	0.41	80%
The operating environment is optimized by				
Maintaining the patient's body temperature	P	5.00	0.00	100%
Checking that the ventilation in the OR is working	P	4.95	0.21	96%
Basic hygiene routines being followed by the OR team	P	4.95	0.21	96%
Covering surgical instruments with a sterile drape after setting them up	P	4.95	0.21	96%
Entire OR team wearing special clothes with low source strength when ventilation in OR requires it	P	4.95	0.22	95%
PRISS‐recommendations (prosthetic‐related infections should stop) being followed during joint replacement surgery	P	4.95	0.21	96%
Entire OR team wearing a surgical hood and a surgical mask during the procedure	P	4.91	0.29	91%
Having the OR closed during an ongoing procedure	P	4.90	0.31	90%
Furnishing the OR so ventilation is optimized	P	4.86	0.35	86%
Surgical instruments not being uncovered until the start of the procedure	P	4.86	0.35	86%
Surgery sensitive to infection (e.g. joint replacement surgery) being planned in an OR with ventilation that ensures the preferred colony forming unit (CFU) level	P	4.86	0.35	86%
Skin antisepsis taking place with Chlorhexidine 5 mg/mL	P	4.82	0.39	82%
Adjusting the number of people in the OR according to the ventilation	P	4.82	0.39	82%
Setting up surgical instruments taking place with closed doors and no patient in the OR	P	4.77	0.53	82%
Sterility during the procedure is ensured by				
Ensuring that the surgical drapes are intact throughout the procedure	P	4.95	0.21	96%
Everyone in the OR team is helping to point out the risks of broken sterility	P	4.95	0.21	96%
Operating room nurse overseeing the entire OR team's activities during the procedure	P	4.86	0.35	86%
Reducing the risk of contamination by keeping clean and dirty surgical instruments separate, e.g. intestinal surgery	P	4.77	0.53	82%
To ensure that the right implant is operated in				
Both surgeon and operating room nurse should read the implant packaging before opening the implant to the sterile field	P	4.82	0.50	86%
Designation on the implant should be compared with the trial prosthesis before the implant is inserted	P	4.75	0.64	85%
For the management of indwelling urinary catheters (IDCs) to be safe in OR				
There need to be a functioning aseptic routine for inserting an IDC	S	4.91	0.29	91%
IDC is inserted by staff with the required competence	S	4.91	0.29	91%
IDC is inserted according to existing routines before the start of the procedure	P	4.82	0.39	82%
IDC is taped to prevent pulling the IDC during the procedure if positioning of the patient poses a risk of pulling	P	4.82	0.39	82%
Scanning of the bladder is performed to avoid insertion of IDC when not needed	P	4.77	0.53	82%
To ensure the safe handling of surgical specimens during the procedure				
There needs to be a well‐defined procedure for specimen management that is followed	S	4.95	0.21	96%
Specimen management needs to be carried out with utmost care to avoid sample contamination	P	4.82	0.50	86%
A new sample needs to be taken if contamination is suspected	P	4.82	0.50	86%
Everyone involved in specimen management in the OR needs to know the procedure	S	4.77	0.53	82%
To ensure the safe positioning of the patient on the OR bed				
Everyone in the OR team needs to help each other in the *advanced* positioning of the patient	P	4.91	0.29	91%
Marks after positioning (e.g. from different supports) need to be reported to staff at the post‐operative ward and noted in the patient's medical record	P	4.91	0.29	91%
Everyone in the OR team needs to know how to avoid injury when positioning the patient	S	4.85	0.37	85%
Everyone in the OR team needs to take responsibility for attaching supports (e.g. side support, hip support) so that it does not come off during the procedure—the person who attaches the support ensures that it is properly attached	P	4.85	0.37	85%
Everyone in the OR team ensures that the patient remains on the OR bed during the procedure	P	4.82	0.39	82%
To ensure that electrosurgical units do not injure the patient during the procedure				
The location of the dispersive electrode needs to be checked after the procedure	P	4.86	0.35	86%
Any contraindication to the use of the electrosurgical unit and settings needs to be checked before the start of the procedure	P	4.86	0.35	86%
All personnel handling electrosurgical units need to be well introduced to the surgical modalities	S	4.85	0.49	90%
To ensure follow‐up and the possibility of improvement				
Pressure injuries suspected to have occurred during the procedure are reported back from the ward staff to the OR staff	P	4.91	0.29	91%
Nerve damage suspected to have occurred during the procedure is reported back from the ward staff to the OR staff	P	4.91	0.29	91%
Problems with postoperative dressings are fed back from the ward staff to the OR staff	P	4.91	0.29	91%
There needs to be a requirement to report back injuries that can be related to the time of surgery to the OR staff	P	4.91	0.29	91%

*Note*: *N* = 73 statements, S = structure indicator, P = process indicator.

The mean for the 30 statements that did not reach consensus ranged from 3.00 to 4.75 (SD 0.44–1.35). These statements are presented in Table 4.

**TABLE 4:** Statements which did not reach consensus (<80%) for all three rounds, including Indicator type, mean, standard deviation and consensus level.

	Indicator type	Mean	SD	Consensus level
For surgical planning to contribute to safe care, it is necessary that				
The planning agrees with the planning on x‐rays (when available)	P	4.40	0.75	55%
The correct operation code has been selected	P	4.35	0.74	45%
A change of surgeon is communicated	P	4.20	0.62	30%
To complete the list of operations scheduled and not have to cancel any				
The implant carts be easy to overview	S	4.00	0.79	25%
There should be an extra OR team outside the OR if there is no room for setting up surgical instruments	S	3.45	0.94	15%
To ensure that the work intraoperatively proceeds optimally				
Surgical instruments and implants should be prepared for possible alternative surgical methods in complex surgery	P	4.70	0.47	70%
A standardized way of working should be followed whenever possible	P	4.50	0.51	50%
Personnel changes should be avoided during an ongoing procedure	S	4.40	0.60	45%
Operating room nurse needs to be able to avoid setting up surgical instruments during the procedure, during the operation itself	P	4.30	0.66	40%
Operating room nurse needs to have time to see the patient before starting preparations	S	4.00	0.56	15%
The flow of information contributes to increased safety during the procedure by				
Reporting to the staff at the postoperative ward taking place systematically	P	4.75	0.44	75%
Implant information is scanned into the medical record—not keyed by hand	S	4.65	0.49	65%
All documentation taking place in the same medical record (e.g. allergies and 0 CRP)	S	4.60	0.68	70%
Reporting taking place systematically in the event of staff replacement (e.g. according to SBAR)	P	4.60	0.50	60%
There is no double documentation	P	4.25	0.79	40%
The use of the Surgical Safety Checklist (SSC) contributes to improved patient safety during the procedure by				
Events that may increase the risk of postoperative infection are also addressed at check‐out	P	4.70	0.47	70%
The operating environment is optimized by				
Keeping the operating time as short as possible	P	4.65	0.59	70%
Personnel changes are avoided during an ongoing procedure	P	4.65	0.49	65%
Furnishing the OR to minimize movement	P	4.55	0.51	55%
Avoid setting up additional surgical instruments during the procedure other than in the event of unforeseen events	P	4.35	0.67	45%
Warming blanket (e.g. Bair hugger®) is started after the surgical draping is completed	P	3.85	1.35	45%
Skin antisepsis taking place with coloured Chlorhexidine 5 mg/mL	P	3.00	1.17	10%
Sterility during the procedure is ensured by				
Reducing the risk of perforation and contamination of surgical gloves by changing gloves, after a certain time or before certain moments during the procedure	P	4.30	0.92	55%
For the management of indwelling urinary catheters (IDCs) to be safe in OR				
Aseptic technique for IDC insertion is used—the catheter is kept sterile when inserted into the bladder	P	4.60	0.60	65%
IDC must be checked according to existing routines before the start of the procedure (if the patient receives the IDC before coming to OR), that there is urine	P	4.60	0.60	65%
It must be clear who prescribes IDC	P	4.25	0.72	40%
To ensure the safe handling of surgical specimens during the procedure				
Handling of specimens needs to be prioritized even when it takes place in relation to the procedure continuing	P	4.70	0.57	75%
Test referrals need to be prepared before the start of the procedure	P	4.10	0.55	20%
To ensure the safe positioning of the patient on the OR bed				
The patient's leg needs to be lifted to see how much it can be lifted if lifting devices are used	P	4.70	0.57	75%
To ensure that electrosurgical units do not injure patient during the procedure				
The location of the dispersive electrode needs to be noted in the patient record	P	4.75	0.44	75%

*Note*: *N* = 30 statements, S = structure indicator, P = process indicator.

## 5 DISCUSSION

The modified Delphi process succeeded in identifying and reaching a consensus on several indicators of perioperative patient safety. Within this group of experts, a total of 73 statements were perceived as very important indicators of perioperative patient safety. Out of these indicators, 74% were process indicators measuring care activity performance, encompassing factors such as compliance with guidelines. At the same time, 26% were structure indicators measuring the foundational conditions, including essential resources such as equipment and staffing levels that facilitate care.

Assessing the quality of a care process necessitates determining if practices important for the outcome are followed. Additionally, it requires that central organizational structures influencing the outcome are established. This link between either established structure or practice and the definitive outcome demands validation either through scientific evidence or consensus by peers (Pronovost et al., 2004). Although most safety indicators revealed in this study were process indicators, several structure indicators were perceived by the experts as very important. One such structure indicator was the statement ‘For surgical planning to contribute to safe care, it is necessary that the right surgical instruments are available’ with a mean score of 4.95 and a standard deviation of 0.21. Similarly, the statement ‘For surgical planning to contribute to safe care, it is necessary that the right implants are available’ had a mean score of 5.00 and a standard deviation of 0.00. Therefore, despite the focus on process indicators in this study, certain structural indicators received notable attention from the experts. These structural indicators are crucial not only for the effective functioning of organizations but also for individual patient outcomes, underscoring the need for their measurement.

In the process of developing indicators in healthcare organizations, the underlying assumption often entails that the best way to ensure overall quality is to measure every aspect of the care provided (Marang‐van de Mheen & Vincent, 2023). However, an alternative perspective could involve assessing the foundational structures, considering how many indicators are needed to reasonably assess the underlying construction. In the present study, two statements highlighted the significance of professionals possessing the right skills and being present during surgical procedures, underscoring the essential prerequisites for ensuring patient safety within the OR. These two statements, ‘To ensure that the work intraoperatively proceeds optimally, all professionals in the OR team need to have the skills required’ and ‘To ensure that the work intraoperatively proceeds optimally, all professionals required need to be in the OR during the procedure’, received mean scores of 4.95 and 4.85 and with standard deviations of 0.21 and 0.35, respectively. Their paramount significance within the OR setting makes them fundamental prerequisites for performing surgical procedures, as their absence could jeopardize patient safety. This refers to the WHO's Global Patient Safety Action Plan 2021–2030, which promotes patient safety measures such as ensuring adequate and competent staffing in the operational plan (WHO, 2021).

Notably, discussions about professional skills often underestimate their important role in upholding the quality of patient care. Professionals are sometimes narrowly viewed as mere executors of processes, inadvertently overlooking their role as individuals in the position to capture critical aspects in certain situations, deserving trust in their professional judgement (Bornemark, 2018). This is where the significance of the Safety II concept lies (Verhagen et al., 2022), which highlights the recognition of uncertainties and the adaptability needed to enhance safety margins, particularly when confronted with daily production pressures. Moreover, the Safety II approach offers valuable insight into the competencies needed to create improved safety margins, aligning them with the complexity of daily work in healthcare organizations. Consequently, ensuring safety within the OR encompasses a comprehensive approach including anticipation, careful planning, and detailed preparation (Göras et al., 2020). To navigate dynamic situations, OR personnel need to possess both experience and the skills needed to coordinate and confirm information. These elements collaboratively facilitate the interpretation of diverse and dynamic situations. Therefore, the presence of skilled OR teams is crucial, facilitating this interpretation and ensuring patient safety during procedures in the OR.

Several indicators revealed in this study align with those in a previous study of core elements in perioperative nursing (Rauta et al., 2013), specifically the ones under the safety dimension. Examples include the perioperative positioning of patients and managing specimens perioperatively. Moreover, six indicators that reached consensus in this study closely resemble the highest‐priority safety issues identified by perioperative nurses (Steelman et al., 2013). As an example, the statement ‘Correct patient and correct side are ensured by confirming indicated side with medical records and x‐rays (when available) before anaesthesia is applied’ in this study and ‘preventing wrong site, wrong procedure, and wrong patient’ in the earlier study. This focus on ensuring the correct patient and side is consistent with the WHO's Global Patient Safety Action Plan, which encourages facility‐level adherence to safety protocols (WHO, 2021). Significantly, the entire expert group in the present study rated the statement as 5 (indicating it is ‘very important’) and the standard deviation is 0, underscoring the robustness of this result.

In many organizations, it appears that what is valued is only what can be easily measured (Bornemark, 2018). This can lead to control activities that risk displacing the organization's core activity, where review and measurement miss targeting meaningful quality work. The indicators regarding teamwork and communication within our findings pose a challenge as they are not easily or readily measurable. However, their importance cannot be undermined as previous research has shown that breakdowns in team communication in the OR were perceived as a notable threat to patient safety (Lingard et al., 2004). Frequently observed breakdowns were that communication occurred too late or was too incomplete to be helpful. In the present study, communication among the team members was seen as very important, with a mean score of 4.95 and a standard deviation of 0.21. Earlier research findings show that successful information exchange needs to be supported by effective communication skills adapted to the present situation (Gillespie et al., 2009).

Unexpectedly, the statement ‘To ensure that the work intraoperatively proceeds optimally, ORN needs to have time to read about the patient before starting preparations’ in our study, only reached consensus in the final round. This finding raises concerns, as the reading about one's patient before the surgical procedure was not initially seen as highly significant by this study's group of experts. One possible explanation could be the influence of workload and competing priorities, leading to reliance on simplified verbal reporting. A previous review study examining documentation practices in ORs revealed the need for enhancing the structure and the technical tools in ORNs' documentation practices (Søndergaard et al., 2017). An absence of a functional cross‐departmental technical platform for documentation may contribute to the low prioritization.

The WHO's Patient Safety Curriculum Guide emphasizes the importance of clear communication among multidisciplinary teams, highlighting its potential to enhance patient care and reduce errors (WHO, 2011). In the present study, effective collaboration and communication within the OR team, and the use of the SSC were considered very important. In this statement group, eight out of nine statements reached consensus in the first round. These aspects were not a part of the findings of previous studies (Steelman et al., 2013; Rauta et al., 2013). However, it is worth noting that Rauta et al.'s study was conducted before the SSC publication by the World Health Organization (WHO) (Rauta et al., 2013).

There is a challenge concerning the delineation of boundaries between ORNs' professional knowledge and the need to establish safety indicators. Healthcare professionals have multiple roles (Smith & Plunkett, 2019), and the primary role is their clinical patient responsibility. Beyond this, they are expected to maintain and improve patient safety in their work and to intervene in the organizational system when needed. Additionally, healthcare professionals are expected to see opportunities for quality improvement and ensure that improvements are made. This approach aligns with WHO's Global Action Plan for Patient Safety (WHO, 2021). The findings of the present study reveal that important perioperative patient safety indicators are part of ORNs' professional knowledge and are interlaced with the skills in their clinical roles. These important indicators, serve as benchmarks for the assessment of perioperative care and place ORNs in a key position in revealing threats to patient safety (Ingvarsdottir & Halldorsdottir, 2018) and they are expected to improve patient safety as an ongoing endeavour.

Many existing indicators tend to focus on effectiveness, safety, and efficiency of care, often overlooking patient‐centred metrics (Chazapis et al., 2018). However, this study has successfully gained consensus on several central perioperative patient safety indicators with a clear patient focus. Notable examples encompass ‘Ensuring correct patient and side being operated’, ‘Ensuring insertion of the correct implant’ and ‘Safe patient positioning’. These indicators hold paramount importance as they not only ensure individual patient safety but also contribute to the organizational learning process. Consequently, it is crucial to assess and closely monitor these indicators.

When constructing a set of indicators, this includes selecting what should be measured trying to avoid redundancy and keeping down the costs of the measurement (Schang et al., 2021). Additionally, there are practical conditions to address, including the involvement of the stakeholders thereby defining the purpose of the measurement. Indicators are used to track quality and safety over time and are used differently depending on what the priorities and purposes are (Marang‐van de Mheen & Vincent, 2023). Therefore, a set of indicators cannot meet the needs of all users equally well. Indicators may be used internally to monitor safety or benchmark care to compare the care to similar institutions. Consequently, if a set of indicators are developed to enable a follow‐up on the ORNs' safety measurement during surgical procedures the indicators must be developed with the ORN group in mind and adapted to meet their needs.

This study identified and defined several indicators intended to reduce avoidable harm among surgical patients, encompassing ORNs' safety measurements that span both care planning and care delivery. Furthermore, these indicators are congruent with many objectives outlined in the World Health Organization's Global Patient Safety Action Plan (WHO, 2021). However, it is important to highlight that the safety indicators defined in this study are not inherently associated with a specific profession. Considering international variations in training and clinical responsibilities of ORNs, certain areas of responsibility may belong to other professions in different countries.

### 5.1 Strengths and limitations

The Delphi approach facilitated the composition of a geographically dispersed expert group of ORNs in clinical practice, involved in management and development, and nursing education and research. This is the significant advantage of an e‐Delphi: a geographically scattered group is combined and rapid feedback can be enabled between the researcher and participants (Keeney et al., 2011). The manageable number of participants included in the study and the close email contact between them, and the researcher contributed to low non‐attrition, which indicates valid and useful findings.

Although only 15% of the experts worked completely clinically, most of them did work clinically to some extent. In the group of ORNs who were partly in leadership roles, most of them worked between 20% and 40% clinically. Similarly, in the group of ORNs in nursing education and research, some of them partly worked clinically. This can be interpreted as this study's experts possess a great deal of clinical experience as leadership and teaching positions are usually occupied by experienced individuals.

The study's focus on the Swedish context may be perceived as a limitation, as ORNs are an established professional group within the OR in this setting. Given the international diversity in the training and clinical roles of ORNs, it is important to acknowledge that in other countries, responsibility for certain indicators or critical process steps may be assigned to other professions. This divergence should be considered when evaluating the applicability and generalizability of the study's findings. Nevertheless, these indicators represent crucial process steps with universal importance, irrespective of the specific profession responsible for their execution.

### 5.2 Recommendations for further research

The findings of this study will provide a foundation for further research endeavours, aimed at refining the current process register in assessing the perioperative process and pinpointing areas where improvement is required. Subsequently, the logical course of action would involve an assessment of the prevailing data collection practices in comparison with the criteria outlined by the experts in this study. This comparative analysis would elucidate the degree of alignment and reveal potential discrepancies, thus informing any necessary adjustments required to align existing data with the indicators identified as crucial.

### 5.3 Relevance to clinical practice/implication for policy and practice

The safety indicators revealed in this study highlight the complexity of patient safety challenges in the OR, necessitating a comprehensive approach that considers procedural aspects and environmental factors. Recognizing the significance of resource management underscores the need for skilled professionals and accessible tools in the OR. The consensus areas provide practical guidance for OR teams to enhance patient safety during surgery. Acknowledging the challenges in measuring factors like teamwork and communication highlights the complexity of addressing these crucial aspects, promoting investment in strategies and training to enhance collaboration and communication within the OR, ultimately benefiting patient safety. This training should be integrated into patient safety training for all healthcare students and staff to capitalize on the benefits of a multidisciplinary team in care improvement and error reduction. Incorporating the indicators into OR nursing specialist education can raise awareness of managing patient safety complexity in the OR and better prepare new ORNs to tackle related challenges.

## 6 CONCLUSION

The safety indicators that reached consensus in this study, designed to assess the perioperative process, underscore the multifaceted challenges involved in ensuring patient safety within the OR environment. These findings imply the compelling need for a balanced integration of structure and process indicators to comprehensively evaluate safety throughout surgical procedures. Further, the study highlighted key areas of consensus, such as the use of the SSC and optimizing the operating room environment. While many of the safety indicators in this study primarily focused on procedural aspects, experts placed high importance on specific structural elements. It is worth noting that the study acknowledges the challenge of continuously measuring factors such as teamwork and communication, which are crucial for ensuring patient safety.

This study provides practical guidance for healthcare practitioners by emphasizing the multifaceted nature of patient safety, the need for a balanced approach to safety assessment, and specific areas of consensus that can be translated into clinical practice to enhance perioperative patient safety.

## AUTHOR CONTRIBUTIONS

All authors conceived the study design. Nyberg Anette collected the data and performed the data analysis, and all authors participated in the data interpretation and manuscript writing and approved the final manuscript version.

## CONFLICT OF INTEREST STATEMENT

No conflicts of interest to declare.

## Data Availability

Responses, analysis and unpublished data from this study are securely stored and only available to AN and can be shared if a reasonable request is submitted to the authors and Umeå University.
